# Microvascular dysfunction in schizophrenia: a case–control study

**DOI:** 10.1038/npjschz.2015.23

**Published:** 2015-07-01

**Authors:** Martin W Vetter, Billie-Jean Martin, Marinda Fung, Milada Pajevic, Todd J Anderson, Thomas J Raedler

**Affiliations:** 1 Department of Psychiatry, Cumming School of Medicine, University of Calgary, Calgery, AB, Canada; 2 Department of Psychiatry, Perelman School of Medicine, University of Pennsylvania, Philadelphia, PA, USA; 3 Department of Cardiac Sciences, Libin Cardiovascular Institute of Alberta, University of Calgary, Calgery, AB, Canada; 4 Hotchkiss Brain Institute, Cumming School of Medicine, University of Calgary, Calgery, AB, Canada

## Abstract

**Background:**

Schizophrenia is a mental illness associated with cardiovascular disease at a younger age than in the general population. Endothelial dysfunction has predictive value for future cardiovascular events; however, the impact of a diagnosis of schizophrenia on this marker is unknown.

**Aims:**

We tested the hypothesis that subjects with schizophrenia have impaired endothelial function.

**Methods:**

A total of 102 subjects (34.5±7.5 years) participated in this study. This sample consisted of 51 subjects with a diagnosis of schizophrenia and 51 healthy subjects, who were matched for age (*P*=0.442), sex (*P*>0.999), and smoking status (*P*=0.842). Peripheral artery microvascular and conduit vessel endothelial function was measured using hyperemic velocity time integral (VTI), pulse arterial tonometry (PAT), and flow-mediated dilation (FMD).

**Results:**

Significantly lower values of VTI were noted in subjects with schizophrenia (104.9±33.0 vs. 129.1±33.8 cm, *P*<0.001), whereas FMD (*P*=0.933) and PAT (*P*=0.862) did not differ between the two groups. A multivariable-linear-regression analysis, built on data from univariate and partial correlations, showed that only schizophrenia, sex, lipid-lowering medications, antihypertensive medications, and low-density lipoprotein (LDL)-cholesterol were predictive of attenuated VTI, whereas age, ethnicity, family history of cardiovascular disease, smoking status, systolic blood pressure, waist circumference, HDL-cholesterol, triglycerides, C-reactive protein, and homeostatic model assessment-insulin resistance (HOMA-IR), antidiabetic medications, antidepressant medications, mood stabilizers, benzodiazepines, and anticholinergic medications did not predict VTI in this model (adjusted *R* ^2^=0.248).

**Conclusions:**

Our findings suggest that a diagnosis of schizophrenia is associated with impaired microvascular function as indicated by lower values of VTI, irrespective of many other clinical characteristics. It might be an early indicator of cardiovascular risk in schizophrenia, and might help to identify high-risk individuals.

## Introduction

Schizophrenia is a severe mental illness that affects ~1% of the population,^1^ and subjects with schizophrenia have a significantly higher burden of cardiovascular disease (CVD)^2–4^ in addition to their psychiatric symptoms. The causes of morbidity and mortality among subjects with schizophrenia follow closely the general population, and heart disease and cerebrovascular incidents accounting for ~50% of deaths.^5^ The life expectancy in schizophrenia is reduced by up to 20 years, with deaths from CVD being far more common than suicide in this population.^5–7^ For instance, the standardized mortality rate from CVD is double in schizophrenia patients compared with those without,^8^ and is particularly elevated in subjects under 50 years of age.^9^ There are some lines of evidence showing that medication side effects and other lifestyle factors do not fully explain the higher burden of cardiovascular morbidity and mortality in schizophrenia patients.^9^

The vascular endothelium is a monolayer of cells that covers the inner surface of blood vessels, and its dysfunction has been implicated in many different disorders.^10,11^ Endothelial dysfunction has been shown to occur before the onset of CVD, making it a potential screening tool^12,13^ and it continues to be recognized as a potential marker of cardiovascular risk.^14,15^ Vascular function has different anatomic compartments; for instance, conduit vessel function represents the macrovasculature, whereas microvascular function is a measure of smaller arteries. Microvascular function can be measured non-invasively via the increase in brachial artery velocity in response to a post-occlusive hyperemic stimulus, the velocity time integral (VTI). VTI has gained acceptance as a surrogate marker of cardiovascular risk in persons without overt CVD^16,17^ and appears to be the optimal measure of microvascular function.^18^ Microvascular function can also be measured using pulse arterial tonometry (PAT),^19^ which may be used in risk stratification of cardiovascular risk.^20^ On the other hand, flow-mediated dilation (FMD) of the brachial artery is the gold standard for the conduit vessel function.^21^

To date, there are no studies that assess peripheral endothelial function via VTI, FMD, and PAT in subjects with schizophrenia. The primary purpose of this study was to compare endothelial function in subjects with schizophrenia and an age-, sex-, and smoking status-matched control group. In view of the significant CVD burden in schizophrenia, we hypothesize that there is evidence of impaired endothelial function in this population, and it might help to identify individuals at particularly high risk.

## Materials and Methods

### Participants

This study was conducted at the University of Calgary, Calgary, AB, Canada. A total of 102 individuals (51 subjects with schizophrenia and 51 controls) between 18 and 45 years of age were recruited for this study. Recruitment was performed with posters within the hospital and outpatient clinics as well as with postings on the Internet. All interested participants were screened over the telephone, and subjects with known CVD, active alcohol and/or substance use, active medical illness, and age above 45 years were excluded from participation. CVD was defined as any history of coronary artery disease, cerebrovascular disease, heart failure, cardiomyopathy, arrhythmia, valvular heart disease, and peripheral vascular disease. As this study focused on a population at risk for the aforementioned conditions, subjects were excluded if overt CVD was present. Subjects with schizophrenia had an illness duration of at least 5 years, which was obtained with patient report and chart review. These subjects were recruited from the Outpatient Schizophrenia Service at the Foothills Medical Centre in Calgary, AB, Canada, and were stabilized with treatment. The diagnosis was primarily made by chart review; however, a physician evaluated all participants with a clinical interview and examination as well. Healthy controls were not allowed to have current or past psychiatric illness or any psychotropic medication use, and they were matched for sex, age, and smoking status. This study was approved by the ethics board of the University of Calgary, and informed consent was obtained from all participants. The recruitment of all participants was conducted from February 2012 to March 2013.

### Baseline assessment

All participants underwent a baseline assessment including a detailed psychiatric and medical history. Demographics included age, sex, ethnicity, duration of illness, and age of onset of schizophrenia, which was validated by chart review in patients with schizophrenia. We used a well-established and validated protocol for the evaluation of cardiac risk factors (hypertension, hypercholesterolemia, diabetes, smoking, and family history of CVD) and other medical conditions (including chronic obstructive pulmonary disease, renal failure, rheumatic disease, inflammatory bowel disease, and valvular heart disease)^16,22^. Again, we inquired about pre-existing CVD, as outlined above, and excluded participants with pre-existing conditions. For patients with schizophrenia, we also cross-referenced their charts for any pre-existing CVD. Again, we asked about drug and alcohol use at that time; however, no samples for drug screening were obtained in this study. We obtained a history about the current antipsychotic medication use (including generic name, estimated start date, and duration of use, lowest dose, maximum dose, average dose) from the participants and by chart review, as well as other medications and supplements. The short form of the International Questionnaire for Physical Activity was previously validated in schizophrenia and was used to quantify the amount of physical activity in both the control and study groups, indicated as metabolic equivalent of task (MET) minutes per week.^23,24^

A physical examination followed, including heart rate, blood pressure, weight, height, body mass index, and waist circumference and hip circumference. A resting electrocardiogram as well as fasting blood work (glucose, creatinine, insulin, hemoglobin, hemoglobin A1c, high-sensitivity C-reactive protein, triglycerides, total cholesterol, low-density lipoprotein (LDL)-cholesterol, and HDL-cholesterol) were obtained for all subjects. Glucose and insulin levels were used to calculate homeostatic model assessment-insulin resistance (HOMA-IR). The presence of metabolic syndrome was defined according to the US National Cholesterol Education Program Adult Treatment Panel III guidelines.^25^

### Assessment of endothelial function

Subjects refrained from eating, smoking, and caffeine ingestion for a minimum of 4 h. We did not measure nicotine or caffeine withdrawal specifically, but we asked the participants about any physical or emotional complaints at the time of study. All vasoactive medications were held for at least 12 h before the scan and blood draw.^26^ The assessment of the endothelial function followed a standardized protocol in a temperature- and noise-controlled environment by a single trained sonographer.^27,28^ The participants were asked to lie on a stretcher and the EndoPAT-probes (Itamar, Israel) were placed on each index finger to measure PAT. The brachial artery was imaged just above the antecubital fossa using a 12-MHz linear phase-arrayed ultrasound transducer and an iE33 ultrasound machine (Philips, Amsterdam, The Netherlands). We measured the PAT and the baseline diameter of the brachial artery and velocity of blood flow via ultrasound. We then assessed the endothelium-dependent FMD. A blood pressure cuff was placed on the right forearm and was inflated to 50 mm Hg above systolic blood pressure for 5 min. Immediately after cuff release we recorded hyperemic velocity with Doppler, and VTI was calculated by tracing the area under the curve (AUC) of the first beat after the release of the cuff. Arterial diameter was sampled at 39 frames/s for 2 min immediately after the Doppler was obtained to determine maximum hyperemic diameter. Analysis of the brachial artery ultrasound images was performed using an automatic edge-detection software (Brachial Image Analyzer, Coralville, IA, USA). The protocols used for calculation of FMD, VTI and PAT are described elsewhere.^26,29^ In short, FMD is calculated as follows: (hyperemic diameter−baseline diameter)/baseline diameter×100%. PAT is calculated using an algorithm that is incorporated in the software. The value is normalized to the control arm and other hemodynamic parameters. During the evaluation of all endothelial markers, the subjects’ arms did not move after a comfortable position was found. Only minimal manipulation occurred while adjusting the ultrasound probe above the antecubital fossa, and there was no movement of the shoulder, elbow, wrist, and fingers.

### Statistical analysis

Variables that are normally distributed are presented as mean±s.d., whereas the median (25th percentile and 75th percentile) was used for non-normally distributed variables (e.g., C-reactive protein). Comparisons between the two groups were conducted using Student’s *t*-test for parametric data, Mann–Whitney *U*-test for nonparametric data, and the *χ* ^2^-test for categorical data. Univariate correlations between parametric data were calculated with Pearson’s product–moment correlation; Spearman’s rank correlation was used for nonparametric and categorical data. Pearson’s partial correlations were used to account for possible confounding factors of the vascular parameters, and to build a backward multiple linear regression model to assess the effects of other cardiovascular risk factors on the measures of endothelial function. The following independent variables were used in the model: diagnosis of schizophrenia, age, sex, ethnicity, family history of CVDe, smoking status, systolic blood pressure, waist circumference, HDL-cholesterol, LDL-cholesterol, triglycerides, C-reactive protein, HOMA-IR, lipid-lowering medication use, antihypertensive medication use, antidiabetic medication use, antidepressant medication use, mood stabilizers, benzodiazepines, and anticholinergic medication use. These variables were selected on the basis of known cardiovascular risk factors, emerging risk factors,^30,31^ and significant findings from the univariate and partial correlation analyses. In order to avoid overfitting, colinear measures (e.g., total cholesterol when LDL-cholesterol is used) were not used.^32^. A two-sided *P* value of less than 0.05 was considered to be significant. IBM SPSS Version 19 (IBM Corporation, Armonk, NY, USA) was used for statistical analysis.

## Results

### Demographic, metabolic characteristics, and medication use

The baseline characteristics of the two study groups (subjects with schizophrenia and controls) were similar across a variety of demographic and clinical characteristics (see Table 1). Six initially enrolled control subjects were excluded from statistical analysis: one participant had a history of recent cocaine use and five controls had a history of depression and/or anxiety treated with antidepressant medications.

Participants were 59% male (*n*=30 in each group) and ranged in age between 19 and 46 years (a 19-year-old patient had had the onset of a psychotic illness at the age of 14, and at the time of study participation this participant was diagnosed with schizophrenia). The mean age for each group was not significantly different (*P*=0.442). The mean duration of schizophrenia was 13.5 years in the study group. Smoking status did not differ between the two study groups (*P*=0.842), but pack years of smoking were different (*P*<0.001). In terms of other cardiovascular risk factors we did not find a difference for hypertension, diabetes (only one patient had a fasting blood glucose ⩾7, but two patients and one control subject had a history of diabetes; for all the participants hemoglobin A1c levels were <6.4), or family history of CVD. However, the mean body mass index and waist circumference were significantly higher in the schizophrenia group (both *P*<0.001) as well as the frequency of dyslipidemia (*P*=0.014).

Differences in medication use were also noted. Patients with schizophrenia were more likely to be using antihypertensive and lipid-lowering medications. All patients with schizophrenia were taking antipsychotic medications; *n*=32 were taking one agent, *n*=17 two agents, and *n*=2 three agents at the time of study. In addition, *n*=14 were taking antidepressant medications, *n*=7 mood stabilizers, *n*=5 benzodiazepines, and *n*=6 anticholinergic medications. Table 2 shows the individual use of antipsychotic medications in detail with the mean dose and duration.

Subjects with schizophrenia demonstrated abnormalities in their metabolic profile (Table 3): compared with control subjects, they had significantly higher values of triglycerides, whereas HDL-cholesterol was significantly lower. There were also differences in glucose metabolism with significantly higher levels of hemoglobin A1c and HOMA-IR. C-reactive protein levels were significantly higher in the schizophrenia group. No significant differences on any other variables, including the amount of weekly physical activity, were demonstrated.

### Vascular assessment

Vascular parameters were different in the schizophrenia and control groups, which can also be seen in Table 3. We found a significant difference in VTI, with a mean value of 104.9 cm in the schizophrenia group and a mean of 129.1 cm in the control group (*P*<0.001). The effect size for the group difference is *d*=0.72. The other two primary vascular end points, FMD and PAT, were not significantly different between the two groups.

In a univariate correlation analysis, we only noted a significant association of a diagnosis of schizophrenia with VTI (*R*=−0.331, *P*<0.001), but not with FMD (*R*=−0.039, *P*=0.697) and PAT (R=−0.032, *P*=0.760). Correlations between the three vascular markers VTI, FMD, and PAT were not significant. VTI was correlated with waist circumference (*R*=−0.204, *P*=0.043), HDL-cholesterol (*R*=0.258, *P*=0.011), and antihypertensive medication use (R=−0.238, *P*=0.016), but not with the other demographic or cardiovascular factors, including the presence of metabolic syndrome, or other cardiac risk-modifying medications. It was also not correlated with specific antipsychotic medications (including duration and mean dose) or other psychotropic medications. The correlation between VTI and antipsychotic medication use did not provide additional information, as all patients with schizophrenia, but none of the control subjects, were taking antipsychotic medications; this was already established with the correlation between a diagnosis of schizophrenia and VTI.

Partial correlations between VTI and a diagnosis of schizophrenia accounting for age, gender, ethnicity, family history of CVD, smoking status (pack years of smoking yielded no different results), systolic blood pressure (diastolic blood pressure yielded no different results), waist circumference, HDL-cholesterol, LDL-cholesterol, triglycerides, C-reactive protein, HOMA-IR, antihypertensive medication use, antidiabetic medication use, lipid-lowering medication use, antidepressant medication use, mood stabilizers, benzodiazepines, and anticholinergic medication use led to significant results for all factors independently. A combined analysis that included all possible confounders was still significant (*R*=−0.336, *P*=0.005). All data from univariate and partial correlations are available in Supplementary Tables.

### Multiple linear regression analysis

Table 4 presents the association of clinical parameters and VTI. A diagnosis of schizophrenia, sex, lipid-lowering medications, antihypertensive medication use, and LDL-cholesterol were the only variables that predicted a lower VTI. Age, ethnicity, family history of CVD, smoking status, systolic blood pressure, waist circumference, HDL-cholesterol, triglycerides, C-reactive protein, HOMA-IR, diabetic medications, antidepressant medications, mood stabilizers, benzodiazepines, and anticholinergic medications did not predict VTI in this model, which explained 24.8% of the variation of VTI (adjusted *R*^2^=0.248).

## Discussion

Endothelial function is a well-established predictor of cardiovascular risk. This study assessed the association between a diagnosis of schizophrenia and a comprehensive set of measures of endothelial function in subjects under the age of 45. Our results indicate that patients with schizophrenia have evidence of microvascular dysfunction on the basis of lower VTI. This association remained significant even after adjusting for other clinical risk factors for CVD. Subjects with schizophrenia and healthy controls were under the age of 45, and were well matched for demographic factors and smoking status. The significant difference in VTI may represent an early surrogate marker for the increased risk for CVD in schizophrenia, and it might be helpful in identifying high-risk individuals. It may also provide more insight into the underlying mechanisms.

There may be several explanations for these findings. Endothelial function is generally divided into microvascular function, measured with VTI and PAT, and macrovascular function, which can be quantified with FMD. VTI is a global marker of microvascular function, in part related to the vascular endothelium. Increasing evidence shows that microvascular dysfunction precedes macrovascular changes.^16,17^ VTI measures an increase in blood flow following a period of ischemia that is mediated by the local reduction in vascular resistance in the blood vessels. VTI can be seen as an integrated measure of microvascular function, anatomically as well as functionally. It is only partially nitric oxide (NO)-dependent, instead of relying on a number of other mediators that are more relevant to the microcirculation. The increase in blood flow, as seen in VTI, causes a higher shear stress in the brachial artery, which subsequently leads to a NO-dependent vasodilation.^33^ Local release of NO by the endothelium drives the dilation of the conduit arteries, and this response is seen ~30 s after cuff release and is mainly dependent on shear stress.^34^ In schizophrenia, there is also evidence of altered NO metabolism in the brain ^35^ and small studies showed an improvement in psychiatric symptoms by infusion of sodium nitroprusside, an exogenous NO donor.^36^ The exact mechanism how NO is implicated in schizophrenia is still under debate; however, exogenous NO seems to improve N-methyl-D-aspartate receptor hypofunction.^36^

PAT is another marker of microvascular function, for which studies noticed concordant changes with VTI.^37^ Martin *et al.* observed a weak relationship between PAT and VTI in a population with diagnosed CVD. In this study we focused on a much younger population without a history of CVD. Changes in endothelial function, as indicated by lower PAT indices may not yet be evident in our study population. Our findings of microvascular endothelial dysfunction, indicated by the lower VTI, may represent a very early disease state in high-risk individuals. An older study population with more pronounced changes in endothelial function might show additional differences in PAT and FMD. Macrovascular endothelial dysfunction is characterized by a diminished vasodilatory response of the brachial artery, and this occurs predominantly in later stages of arteriosclerosis.^16^ Again, the young age of our study population and the lack of manifest CVD may explain the lack of changes in macrovascular endothelial function.

We performed a linear regression model, which showed only schizophrenia, sex, lipid-lowering medication use, antihypertensive medication use, and LDL-cholesterol as predictors of VTI. We did not find an association with the other demographic factors, laboratory markers, and psychotropic medication use, even though some of these factors differed between the control and schizophrenia group as well as there being significant univariate correlation of VTI with waist circumference and HDL-cholesterol. These results suggest that a diagnosis of schizophrenia is a predictor of VTI along with other demographic or clinical factors.

There is evidence that schizophrenia can be a risk factor for insulin resistance and diabetes mellitus^38^, with drug-naive patients showing signs of abnormal glucose metabolism;^39^ however, there is also evidence that unmedicated patients have similar rates of metabolic syndrome compared with the general population.^40,41^ As all patients were medicated with antipsychotics, it is difficult to distinguish the effects of schizophrenia itself from the effects of the medication. There is a myriad of literature on the metabolic effects of antipsychotics,^42^ and there is also some lines of evidence that certain antipsychotic medications impair endothelial function in animal models.^43^ Currently available antipsychotics can adversely affect physical health, as they are associated with an increased incidence of cardiovascular risk factors. Antipsychotics seem to have different pathways of increasing cardiovascular mortality. On the one hand, vascular disease burden is mediated through long-term effects of all major risk factors for atherosclerosis, including dyslipidemia and diabetes.^44,45^ Treatment with antipsychotics carries the risk of potentiating the baseline cardiometabolic risk,^46^ even in early stages of psychotic disorders.^47^ We indeed noted differences in the metabolic profiles of patients with schizophrenia. Glucose metabolism was impaired, indicated by higher fasting glucose levels, hemoglobin A1c, insulin levels as well as HOMA-IR values, which is an indicator for basal insulin resistance.^48^ There is evidence that insulin as well as insulin resistance can cause endothelial dysfunction. A study from 2002 found that insulin itself impaired endothelial function, indicated by lower FMD.^49^ There was a difference in frequency of metabolic syndrome between the schizophrenia and control group. The frequency of metabolic syndrome in the schizophrenia group was 29.4%, which is in the same range as findings from other studies.^40,41^ Metabolic syndrome is known to impair mircovascular function, indicated by attenuated VTI.^33^

### Limitations

Our study provides a comprehensive assessment of macro- and microvascular endothelial functions via VTI, PAT, and FMD in subjects with schizophrenia and matched healthy controls to the age of 45. Our findings are consistent with other studies that have found a attenuation of microvascular endothelial function; however, these studies used Laser-Doppler flowmetry, which measures blood flow in the superficial dermal plexus^50,51^ or applied retinal vasculature assessment.^52^ Although both groups were matched for age, sex, and smoking status, we still observed differences in other demographic and clinical parameters. All 51 subjects with schizophrenia in this study were taking antipsychotics at the time of the study, which we could not control for. As there is little literature on the effects of antipsychotic medications on endothelial microvascular dysfunction, it cannot be excluded that this may represent a significant confounding factor. However, analysis of individual antipsychotic medications, including the mean dose and duration, did not identify a particular antipsychotic that is associated with lower VTI value. We relied on patient reports and chart reviews for medication data, and this might explain the possible heterogeneity of the obtained data. Although matching for age, gender, and smoking status occurred, we still found a significant difference in waist circumference and body mass index. There was a significant univariate correlation between VTI and waist circumference, HDL-cholesterol, and antihypertensive medication use; however, the connection between a diagnosis of schizophrenia and VTI in partial correlations and multivariable analysis remained significant. Still, obesity (as indicated by higher waist circumferences) and low HDL-cholesterol could be a link between antipsychotic use and impaired endothelial function. Therefore, future longitudinal studies with drug-naive subjects may help to disambiguate the effect of schizophrenia itself and drug effects, and if endothelial function changes over time in course of illness and with drug therapy. Furthermore, prospective studies are needed to establish whether impaired endothelial function increases risk for CVD in schizophrenia later in life. Participants in this study were no older than 45 years. This age limit was chosen to examine a population at risk for CVD, but with no overt manifestations. However, this cutoff might limit the generalizability of the results to different age groups.

### Implications

The present study elicited novel information on the vascular function in patients with schizophrenia. VTI, a simple measure of microvascular function, was significantly lower in this population with a fairly large difference in effect size. There is increasing literature that supports that VTI is a useful surrogate marker in cardiovascular risk assessment and it helps to classify individual risk.^16^ As VTI is an easy and reliable marker of vascular function, it may be a useful marker of cardiovascular risk. Whereas VTI has shown to offer prognostic information in certain populations without mental illness,^16^ it needs to be evaluated in prospective studies in psychiatric populations. It must also be determined whether therapeutic interventions on the basis of changes in vascular function will be beneficial. Our findings may help to identify subjects at high risk for cardiovascular complications. A recent study from Sweden shows that subjects with schizophrenia were less likely to be diagnosed with CVD before fatal cardiovascular events.^7^

## Conclusion

This study shows that peripheral microvascular dysfunction is present in patients with schizophrenia under the age of 45. The two cohorts of subjects with schizophrenia and healthy controls were similar in many demographic characteristics. This study adds to the increasing body of evidence of abnormal vasculature in schizophrenia^53,54^ and schizophrenia being a systemic disorder, affecting multiple organs and systems.^55,56^ The difference in VTI may be an early indication of their cardiovascular risk. Mental illness has a significant burden on the somatic health of individuals and we need to better understand the interface of mental and physical health.

## Figures and Tables

**Table 1:** Baseline characteristics of study subjects

	*Schizophrenia; N (%)*	*Control; N (%)*	P-*value*
Total sample	51	51	—
Male	30 (58.8)	30 (58.8)	>0.999
Female	21 (41.2)	21 (41.2)	>0.999
Caucasian ethnicity	33 (64.7)	38 (74.5)	0.110
			
*Cardiovascular risk factors*			
Hypertension	2 (3.9)	0 (0)	0.160
Diabetes	2 (3.9)	1 (2.0)	0.558
Dyslipidemia	10 (19.6)	2 (3.9)	0.014
Current smokers	23 (45.1)	22 (43.1)	0.842
Family history of CVD	20 (39.2)	19 (37.2)	0.839
Metabolic syndrome (ATP III definition)^a^	15 (29.4)	3 (5.9)	0.002
			
*Psychotropic medications*			
Antipsychotic medication use	51 (100)	n/a	
Antidepressant medication use	14 (27.4)	n/a	—
Mood stabilizers	7 (13.7)	n/a	—
Benzodiazepines	5 (9.8)	n/a	—
Anticholinergic medication use	6 (11.8)	n/a	—
			
*Other medications*			
Antihypertensive medication use	6 (11.8)	0 (0)	0.012
Lipid-lowering medication use	9 (17.7)	0 (0)	0.002
Antidiabetic medication use	2 (3.9)	0 (0)	0.160

	*Mean*	*s.d.*	*Mean*	*s.d.*	
Age (years)	35.1	7.1	33.9	7.9	0.442
Disease duration (years)	13.5	6.8	n/a	—	
Mean body mass index (kg/m^2^)	30.7	7.5	24.9	4.7	<0.001
Waist circumference (cm)	101.0	15.0	80.6	12.0	<0.001
Systolic blood pressure (mm HG)	114.5	11.9	117.7	12.5	0.186
Diastolic blood pressure (mm HG)	75.2	8.6	73.9	8.1	0.443
Pack years of smoking	7.32	8.117	2.259	4.302	<0.001

	*Median*	*25th, 75th percentile*	*Median*	*25th, 75th percentile*	
Total physical activity (MET*min/week)	1,830	(1,094, 3,545)	2,680	(1,201, 7,011)	0.115

Abbreviations: ATP III, Adult Treatment Panel III; cardiovascular disease cardiovascular disease; MET, metabolic equivalent of task.

a ATP III definition (three of five required): waist circumference >102 cm for males, >88 cm for females; blood pressure ⩾130/85 mm Hg; HDL<1.04 mmol/l for males, <1.29 for females; triglycerides ⩾1.7 mmol/l; fasting glucose ⩾6.1 mmol/l.

**Table 2:** Antipsychotic medication use

*Antipsychotic*	N *(%)*	*Mean dose (mg)*	*s.d.*	*Mean duration (months)*	*s.d.*
Clozapine	21 (41.2%)	336.0	160.0	53.1	16.4
Risperidone	11 (21.6%)	2.9	1.4	38.6	25.2
Aripiprazole	10 (19.6%)	14.2	8.2	10.3	9.4
Olanzapine	7 (13.7%)	18.9	11.5	42.2	19.7
Quetiapine	7 (13.7%)	435.7	409.9	21.8	19.3
Paliperidone	6 (11.8%)	8.0	6.0	22.2	15.3
Ziprasidone	4 (7.8%)	67.5	18.9	29.8	18.4
Zuclophenthixol	3 (5.9%)	13.8	5.6	60	n/a
Asenapine	1 (2.0%)	5.0	n/a	3.0	n/a
Haloperidol	1 (2.0%)	2.0	n/a	60	n/a
Loxapine	1 (2.0%)	n/a	n/a	n/a	n/a

Abbreviation: n/a, not available.

**Table 3:** Vascular and metabolic parameters

	*Schizophrenia (N)*		*Control (N)*		P-*value*
Total sample	51		51		—
	*Mean*	*s.d.*	*Mean*	*s.d.*	
*Vascular parameters*					
Velocity time integral (cm)	104.9	33.0	129.1	33.8	<0.001
Flow-mediated dilation (%)	7.1	4.8	7.1	3.8	0.933
Pulse arterial tonometry	1.94	0.58	1.92	0.43	0.862
					
Baseline diameter of brachial artery (cm)	4.06	0.671	3.97	0.636	0.488
					
*Metabolic parameters*					
Total cholesterol (mmol/l)	4.72	0.82	4.37	0.73	0.028
HDL-cholesterol (mmol/l)	1.15	0.30	1.49	0.46	<0.001
LDL-cholesterol (mmol/l)	2.71	0.79	2.38	0.69	0.033
Glucose fasting (mmol/l)	5.24	1.07	4.79	0.36	0.007
Hemoglobin A1_c_ (%)	5.65	0.29	5.39	0.28	<0.001
Hemoglobin (g/l)	148	14	150	13	0.415

	*Median*	*25*^*th*^ *, 75*^*th*^ *percentile*	*Median*	*25*^*th*^ *, 75*^*th*^ *percentile*	
Creatinine (μmol/l)	77	21	77	15	0.890
High-sensitivity C-reactive protein (mg/l)	1.50	(0.70, 3.22)	0.80	(0.40, 1.22)	0.003
Insulin (pmol/l)	58.9	(42.9, 96.4)	34.8	(22.6, 42.6)	<0.001
HOMA-IR	1.82	(1.25, 3.36)	1.04	(0.66, 1.34)	<0.001
Triglycerides (mmol/l)	1.61	(1.11, 2.57)	0.88	(0.67, 1.21)	<0.001

Abbreviations: HDL, high-density lipoprotein; HOMA-IR, homeostatic model assessment-insulin resistance; LDL, high-density lipoprotein.

**Table 4:** Best fitting linear regression model for velocity time integral (*n*=87)

*Predictors*	*Beta*	P-*value*
Schizophrenia	−0.416	<0.001
Male sex	−0.290	0.004
Lipid-lowering medication use	0.261	0.015
Antihypertensive medication use	−0.220	0.029
LDL-cholesterol	0.207	0.038

Abbreviations: HDL, high-density lipoprotein; HOMA-IR, homeostatic model assessment-insulin resistance; LDL, low-density lipoprotein.

Adjusted *R*^2^=0.248; variables excluded by model: age, ethnicity, family history of cardiovascular disease, smoking status, systolic blood pressure, waist circumference, HDL-cholesterol, triglycerides, C-reactive protein, HOMA-IR, antidiabetic medication use, antidepressant medication use, mood stabilizers, benzodiazepines, and anticholinergic medication use.
