# Supplementary material for: Microvascular dysfunction in schizophrenia: a case–control study
Source: NPJ Schizophr. 2015 Jul 1;1:15023–. doi: 10.1038/npjschz.2015.23 (PMC4849449; doi:10.1038/npjschz.2015.23)
Supplement: Supplementary Information [file npjschz201523-s1.doc]

**Table 1: Univariate Correlations**

| **Marker** | **Flow-mediated dilation** | **Velocity Time Integral** | **Pulse Arterial Tonometry** |
| --- | --- | --- | --- |
|  | **Correlation-coefficient (p-value)** | **Correlation-coefficient (p-value)** | **Correlation-coefficient (p-value)** |
| **Flow-mediated dilation** |  | .136  (0.172) | -.149  (.153) |
| **Velocity Time Integral** | .136  (.172) |  | .163  (.119) |
| **Pulse Arterial Tonometry** | -0.149  (.153) | 0.163  (0.119) |  |
| **Diagnosis of schizophrenia** | -.039  (.697) | **-.331**  **(<.001)** | -.032  (.760) |
| **Male Sex** | -.005  (.957) | -.180  (.070) | **-.364**  **(<.001)** |
| **Ethnicity** | -.018  (.859) | -.004  (.966) | .015  (.891) |
| **Age** | -.070  (.485) | .107  (.281) | .185  (.076) |
| **Body Mass Index** | -.058  (.569) | -.125  (.212) | .123  (.244) |
| **Waist circumference** | -.010  (.925) | **-.204**  **(.043)** | -.127  (.232) |
| **Hip circumference** | .014  (.888) | -.099  (.330) | .049  (.647) |
| **Systolic blood pressure** | -.114  (.255) | -.014  (.889) | -.095  (.535) |
| **Diastolic blood pressure** | -.038  (.707) | -.061  (.548) | **-.205**  **(.048)** |
| **Total cholesterol** | .004  (.968) | .065  (.525) | -.069  (.524) |
| **HDL cholesterol** | .172  (.091) | **.258**  **(.011)** | .195  (.068) |
| **LDL cholesterol** | -.024  (.817) | .052  (.616) | -.051  (636) |
| **Triglycerides** | -.176  (.085) | -.169  (.098) | -.189  (.077) |
| **Fasting glucose** | -.112  (.272) | -.039  (.709) | -.209  (.051) |
| **Hemglobin A1c** | -.084  (.425) | -.172  (.101) | -.041  (.712) |
| **Insulin levels** | -.118  (.266) | -.196  (.063) | -.163  (.143) |
| **Homeostatic model assessment – insulin resistance** | -.118  (.263) | -.204  (.053) | -.186  (.094) |
| **C-reactive protein levels** | .014  (.895) | -.070  (.521) | -.121  (.283) |
| **Current smokers** | .119  (.232) | -.079  (.427) | -.195  (.061) |
| **Pack years of smoking** | .020  (.847) | -.166  (.107) | -.131  (.230) |
| **Hypertension by patient report** | .037  (.717) | -.044  (.663) | .000  (>.999) |
| **Dyslipidemia by patient report** | **.311**  **(.001)** | .026  (.797) | -.087  (.409) |
| **Diabetes mellitus by patient report** | .038  (.701) | -.034  (.738) | -.055  (.599) |
| **Family history of coronary artery disease** | .057  (.573) | .046  (.644) | -.010  (.926) |
| **Metabolic Syndrome ^a)^** | -.056  (.577) | -.096  (.337) | -.156  (.135) |
| **Total physical activity (MET*min/week)** | -.198  (.061) | .007  (.944) | .070  (.535) |
| **Antihypertensive medication use** | .191  (.054) | **-.238**  **(.016)** | **-.262**  **(.011)** |
| **Lipid-lowering medication use** | **.254**  **(.010)** | .041  (.686) | .016  (.882) |
| **Antidiabetic medication use** | -.031  (.755) | -.061  (.541) | -.159  (.127) |
| **Antipsychotic medication use** | -.039  (.697) | **-.331**  **(<.001)** | -.032  (.760) |
| **Antidepressant medication use** | .008  (.934) | -.079  (.429) | .032  (.760) |
| **Mood stabilizer use** | -.076  (.445) | -.173  (.082) | -.006  (.957) |
| **Benzodiazepine use** | .181  (.068) | -.135  (.176) | -.074  (.483) |
| **Anticholinergic medication use** | -.062  (.534) | -.078  (.437) | -.046  (.660) |
| **Clozapine use** | .055  (.699) | -.119  (.405) | -.272  (.070) |
| **Clozapine mean dose (mg)** | -.063  (.790) | -.006  (.979) | .246  (.342) |
| **Clozapine duration (months)** | .245  (.343) | .018  (.944) | .242  (.405) |
| **Olanzapine use** | -.058  (.686) | -.112  (.433) | .035  (.818) |
| **Olanzapine mean dose (mg)** | .393  (.383) | .214  (.645) | .029  (.957) |
| **Olanzapine duration (months)** | -.395  (.439) | -.516  (.295) | -.051  (.935) |
| **Aripiprazole use** | -.101  (.482) | .091  (.527) | -.009  (.953) |
| **Aripiprazole mean dose (mg)** | -.003  (.993) | .179  (.621) | -.170  (.688) |
| **Aripiprazole duration (months)** | -.593  (.092) | -.371  (.325) | .364  (.423) |
| **Quetiapine use** | .008  (.957) | -.118  (.409) | .064  (.677) |
| **Quetiapine mean dose (mg)** | -.144  (.758) | -.108  (.818) | -.054  (.908) |
| **Quetiapine duration (months)** | .100  (.873) | -.500  (.391) | -.600  (.285) |
| **Paliperidone use** | .099  (.488) | .236  (.096) | .**307**  **(.040)** |
| **Paliperidone mean dose (mg)** | .205  (.741) | -.462  (.434) | -.051  (.935) |
| **Paliperidone duration (months)** | .359  (.553) | .718  (.172) | -.359  (.553) |
| **Zuclopenthixol use** | -.102  (.477) | -.028  (.844) | -.062  (.684) |
| **Zuclopenthixol mean dose (mg)** | -.500  (.667) | .500  (.667) | n/a |
| **Zuclopenthixol duration (months)** | n/a | n/a | n/a |
| **Risperidone use** | **.279**  **(.048)** | .099  (.490) | -.165  (.280) |
| **Risperidone mean dose (mg)** | .468  (.147) | -.455  (.159) | -.492  (.148) |
| **Risperidone duration (months)** | **.736**  **(.038)** | .342  (.406) | .149  (.750) |
| **Ziprasidone use** | -.159  (.266) | -.166  (.244) | .079  (.606) |
| **Ziprasidone mean dose (mg)** | .105  (.895) | -.316  (.684) | -.866  (.333) |
| **Ziprasidone duration (months)** | .800  (.200) | .000  (1.000) | -.500  (.667) |
| **Loxapine use** | .115  (.420) | -.096  (.502) | .186  (.222) |
| **Loxapine mean dose (mg)** | n/a | n/a | n/a |
| **Loxapine duration (months)** | n/a | n/a | n/a |
| **Haloperidol use** | -.221  (.119) | -.240  (.089) | .139  (.361) |
| **Haloperidol mean dose (mg)** | n/a | n/a | n/a |
| **Haloperidol duration (months) (months)** | n/a | n/a | n/a |
| **Asenapine use** | .077  (.592) | .173  (.225) | -.023  (.880) |
| **Asenapine mean dose (mg)** | n/a | n/a | n/a |
| **Asenapine duration (months)** | n/a | n/a | n/a |
| a) ATP III definition (3 of 5 required): Waist circumference > 102cm for males, > 88cm for females; Blood pressure ≥ 130/85mmHg; HDL < 1.04mmol/L for males, < 1.29 for females, Triglycerides ≥ 1.7mmmol/L, fasting glucose ≥ 6.1mmol/L | | | |

**Table 2: Partial Correlations**

| **Marker** | **Flow-mediated dilation** | **Velocity Time Integral** | **Pulse Arterial Tonometry** |
| --- | --- | --- | --- |
|  | **Correlation-coefficient (p-value)** | **Correlation-coefficient (p-value)** | **Correlation-coefficient (p-value)** |
| **Diagnosis of schizophrenia** | -.008  (.950) | **-.336**  **(.005)** | .162  (.209) |

The following factors were accounted for age, sex, ethnicity, family history of cardiovascular disease, smoking status, systolic blood pressure, waist circumference, HDL-, LDL-cholesterol, triglycerides, C-reactive protein, HOMA-IR, lipid-lowering medication use, antihypertensive medication use, antidiabetic medication use, antidepressant medication use, mood stabilizers, benzodiazepines and anticholinergic medication use.

Table 3: Best fitting linear regression model for Flow-mediated dilation (n=87)

| **Predictors** | **beta** | **p-value** |
| --- | --- | --- |
|  |
| **Lipid-lowering medication use** | .284 | **.010** |
| **Antihypertensive medication use** | .231 | **.029** |
| **HDL-cholesterol** | .219 | .061 |
| **Triglycerides** | -.203 | .091 |

Adjusted R square = 0.193

The following factors were accounted for diagnosis of schizophrenia, age, sex, ethnicity, family history of cardiovascular disease, smoking status, systolic blood pressure, waist circumference, HDL-, LDL-cholesterol, triglycerides, C-reactive protein, HOMA-IR, lipid-lowering medication use, antihypertensive medication use, antidiabetic agents, antidepressant medication use, mood stabilizers, benzodiazepines and anticholinergic medication use.

**Table 4:** Best fitting linear regression model for Velocity Time Integral (n=87)

| **Predictors** | **beta** | **p-value** |
| --- | --- | --- |
|  |
| **Schizophrenia** | -0.416 | **<0.001** |
| **Male sex** | -0.290 | **0.004** |
| **Lipid-lowering medication use** | 0.261 | **0.015** |
| **Antihypertensive medication use** | -0.220 | **0.029** |
| **LDL-cholesterol** | 0.207 | **0.038** |

Adjusted R square = 0.248

The following factors were accounted for diagnosis of schizophrenia, age, sex, ethnicity, family history of cardiovascular disease, smoking status, systolic blood pressure, waist circumference, HDL-, LDL-cholesterol, triglycerides, C-reactive protein, HOMA-IR, lipid-lowering medication use, antihypertensive medication use, antidiabetic medication use, antidepressant medication use, mood stabilizers, benzodiazepines and anticholinergic medication use.

**Table 5: Best fitting linear regression model for Pulse arterial tonometry** (n=81)

| **Predictors** | **beta** | **p-value** |
| --- | --- | --- |
|  |
| **Male sex** | -0.374 | **<0.001** |

Adjusted R square = 0.129

The following factors were accounted for diagnosis of schizophrenia, age, sex, ethnicity, family history of cardiovascular disease, smoking status, systolic blood pressure, waist circumference, HDL-, LDL-cholesterol, triglycerides, C-reactive protein, HOMA-IR, lipid-lowering medication use, antihypertensive medication use, antidiabetic medication use, antidepressant medication use, mood stabilizers, benzodiazepines and anticholinergic medication use.

**Figure 1: Scatterplot between Velocity Time Integral and Diagnosis of Schizophrenia**

**
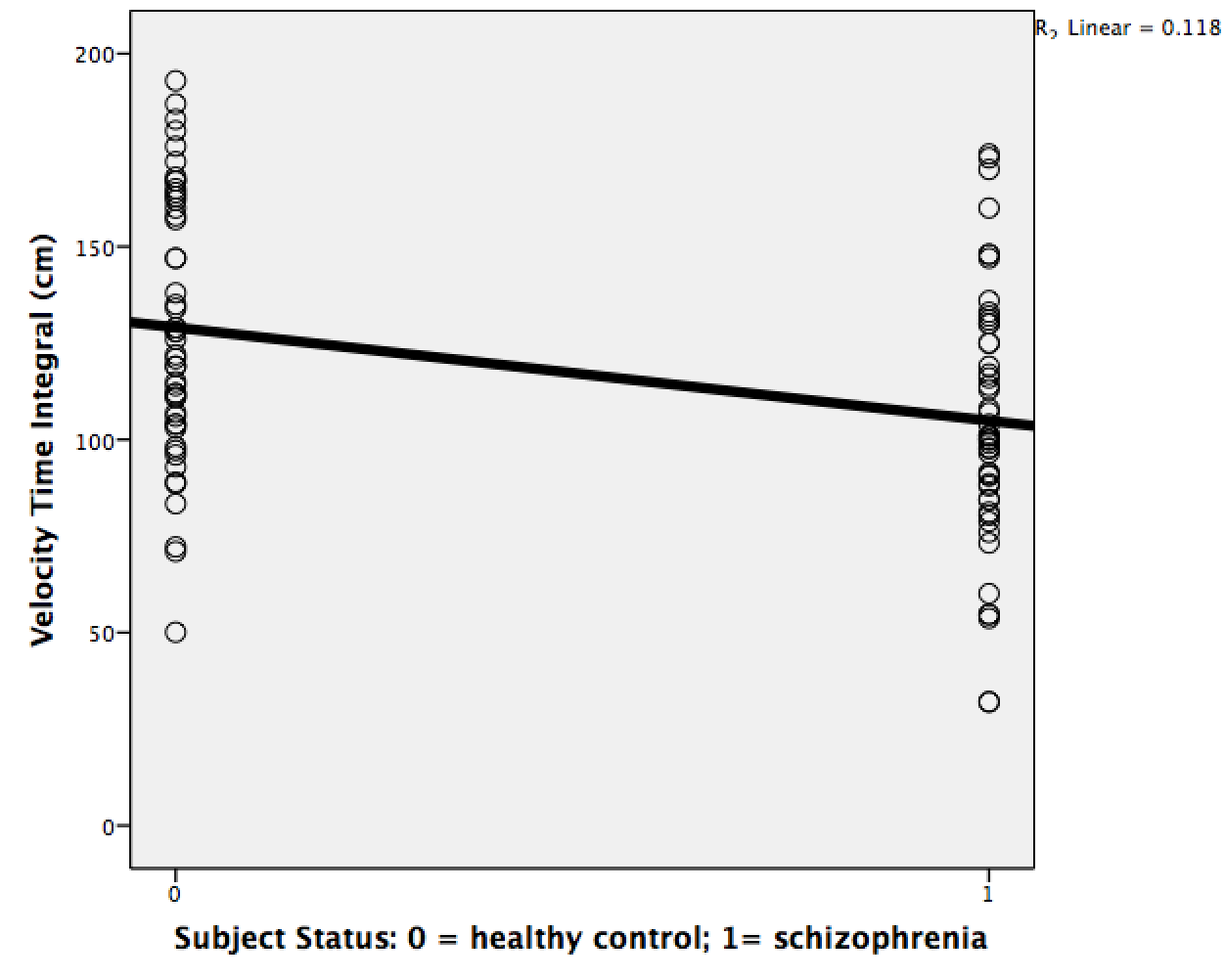
**

**Figure 2: Scatterplot between Velocity Time Integral and waist circumference**

**
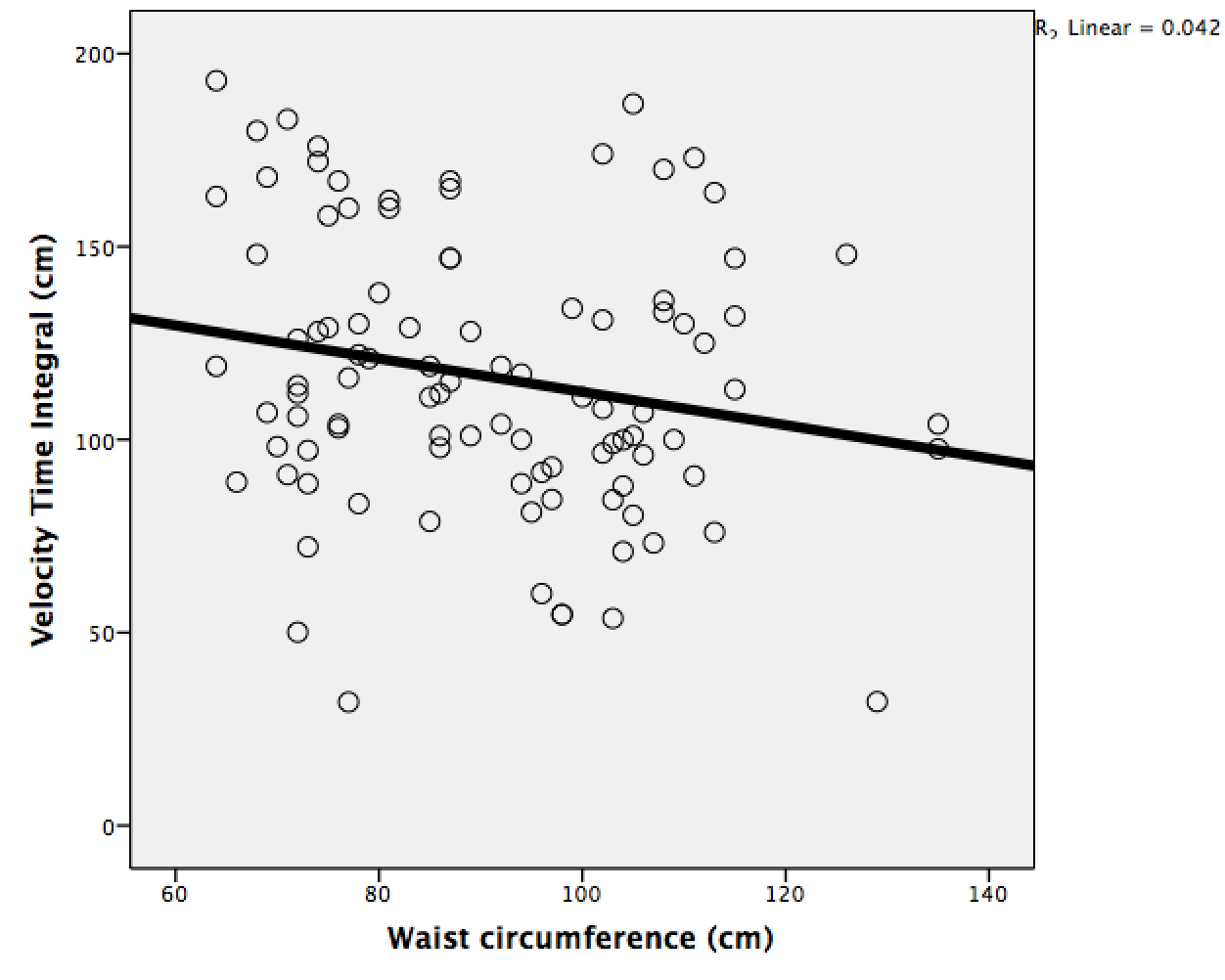
**

**Figure 3: Scatterplot between Velocity Time Integral and HDL-cholesterol**

**
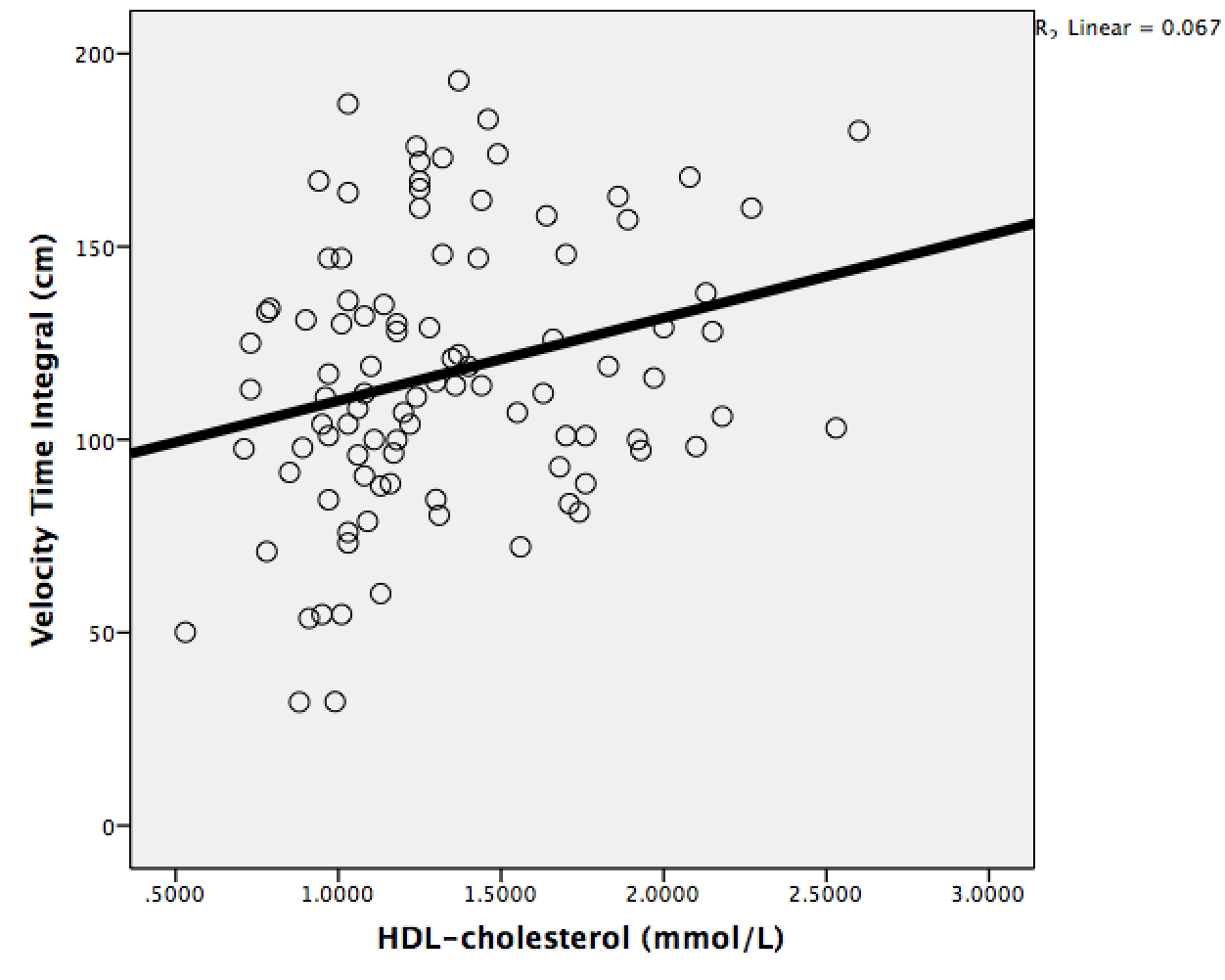
**

**Figure 4: Scatterplot between Velocity Time Integral and Antihypertensive medication use**

**
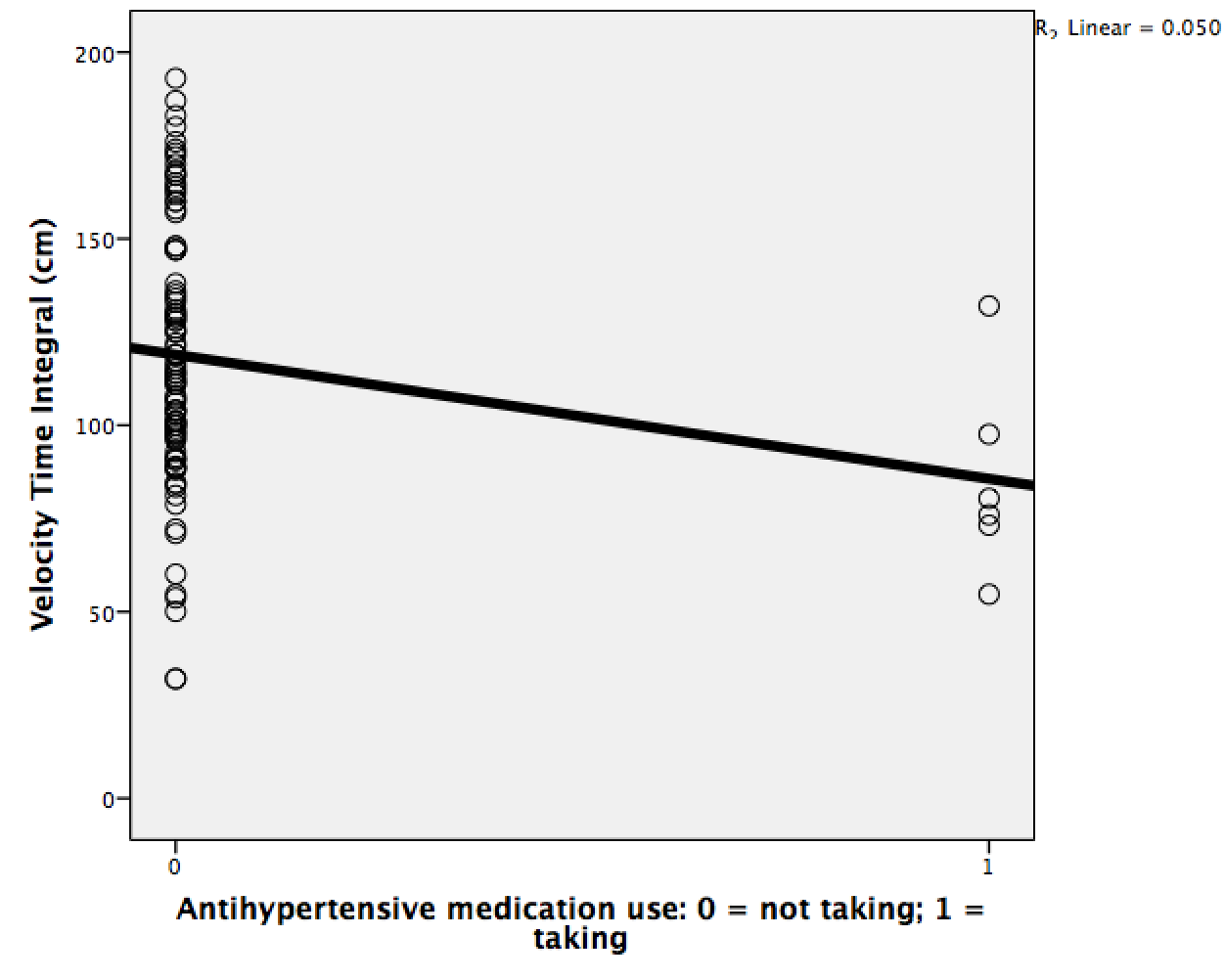
**
